# Kindlin-1 Regulates Epidermal Growth Factor Receptor Signaling

**DOI:** 10.1016/j.jid.2018.08.020

**Published:** 2019-02

**Authors:** Magdalene Michael, Rumena Begum, Grace K. Chan, Austin J. Whitewood, Daniel R. Matthews, Benjamin T. Goult, John A. McGrath, Maddy Parsons

**Affiliations:** 1 Randall Division of Cell and Molecular Biophysics, King’s College London, Guy’s Campus, London, UK; 2 St. Johns Institute of Dermatology, King’s College London, Guy’s Campus, London, UK; 3 School of Biosciences, University of Kent, Canterbury, Kent, UK; 4 Nikon Imaging Centre, King’s College London, Guy’s Campus, London, UK

**Keywords:** EGFR, epidermal growth factor receptor, FRET, fluorescence resonance energy transfer, IP, immunoprecipitation, KS, Kindler syndrome, MST, microscale thermophoresis, PBS, phosphate buffered saline, WT, wild type

## Abstract

Kindler syndrome is an autosomal recessive genodermatosis that results from mutations in the *FERMT1* gene encoding t kindlin-1. Kindlin-1 localizes to focal adhesion and is known to contribute to the activation of integrin receptors. Most cases of Kindler syndrome show a reduction or complete absence of kindlin-1 in keratinocytes, resulting in defective integrin activation, cell adhesion, and migration. However, roles for kindlin-1 beyond integrin activation remain poorly defined. In this study we show that skin and keratinocytes from Kindler syndrome patients have significantly reduced expression levels of the EGFR, resulting in defective EGF-dependent signaling and cell migration. Mechanistically, we show that kindlin-1 can associate directly with EGFR in vitro and in keratinocytes in an EGF-dependent, integrin-independent manner and that formation of this complex is required for EGF-dependent migration. We further show that kindlin-1 acts to protect EGFR from lysosomal-mediated degradation. This shows a new role for kindlin-1 that has implications for understanding Kindler syndrome disease pathology.

## Introduction

Kindler syndrome (KS) (OMIM 173650) is a rare autosomal recessive skin disorder for which there is currently no cure. Genome-wide linkage analysis showed that KS is caused by an abnormality in the actin cytoskeleton and its association with the extracellular matrix due to a deficiency or defect in the focal adhesion protein kindlin-1 (also known as fermitin family homologue 1) (Jobard et al., 2003, Siegel et al., 2003). Clinical features of KS range from trauma-induced blistering, progressive poikiloderma and skin atrophy, photosensitivity, destructive periodontal disease, severe colitis, and squamous cell carcinoma (Ashton, 2004, Lai-Cheong et al., 2007). Since identifying the *FERMT1* gene, at least 170 patients and 60 mutations have been reported. These mutations include nonsense, frameshift splice site, and internal deletion changes all resulting in loss of expression (Has et al., 2011, Techanukul et al., 2011). The human *FERMT1* gene encodes the protein kindlin-1, and other members of this protein family include kindlin-2 and kindlin-3 (Siegel et al., 2003). Although related, these proteins exhibit differential expression patterns: kindlin-1 expression is predominantly restricted to epithelial cells, kindlin-2 is widely expressed, and kindlin-3 is present in hematopoietic and endothelial cells (Bialkowska et al., 2010, Lai-Cheong et al., 2009, Siegel et al., 2003, Wiebe et al., 2008). Both kindlin-1 and kindlin-2 localize to focal adhesions, and kindlin-2 is also recruited to cell-cell junctions (Brahme et al., 2013, Lai-Cheong et al., 2008), whereas kindlin-3 localizes to podosomes (Meves et al., 2009). All kindlins have a bipartite FERM (i.e., 4.1 protein, ezrin, radixin, moesin) domain consisting of four subdomains (F0, F1, F2, and F3) that are present in many proteins involved in cytoskeletal organization (Baines et al., 2014, Goult et al., 2009). The kindlin F2 subdomain differs from other FERM domain proteins by an insertion of a pleckstrin homology (i.e., PH) domain that binds phosphoinositide phosphates (Meves et al., 2009).

Kindlins have all been shown to bind directly to the cytoplasmic domain of β-integrin subunits and contribute to integrin activation (Rognoni et al., 2016). In normal skin, kindlin-1 localizes in basal keratinocytes at the dermal-epidermal junction and accumulates at cell-matrix adhesion sites. In isolated keratinocytes, kindlin-1 localizes to the cell leading edge and focal adhesions (Larjava et al., 2008). Depletion of kindlin-1 leads to reduced proliferation, adhesion, and spreading and to reduced directed migration, with the cells displaying multiple leading edges and multipolar shapes (Has et al., 2008, Herz et al., 2006, Zhang et al., 2016). The role of kindlin-1 in integrin-mediated processes provides explanation for some of the clinical features observed in patients with KS. Potential non–integrin-related roles for kindlin-1 in controlling cell behavior remain unclear.

In this study we performed mass spectrometry analysis of keratinocytes from KS patients and identified significantly reduced levels of the epidermal growth factor receptor (EGFR) in KS samples. Further analysis showed defective downstream signaling of EGFR and attenuated cell responses to EGF stimulation. The expression of kindlin-1 in KS cells was able to restore EGFR expression levels and responses to EGF. Our investigations showed a direct interaction between kindlin-1 and EGFR at the plasma membrane that acts to protect EGFR from lysosomal degradation, independent of kindlin-1 binding to integrins. These data provide new insight into kindlin-1 function in keratinocytes and may provide new avenues for pursuit of therapeutic strategies to treat KS patients.

## Results and Discussion

### KS keratinocytes have reduced levels of EGFR and attenuated response to EGF stimulation

**Figure 1:**
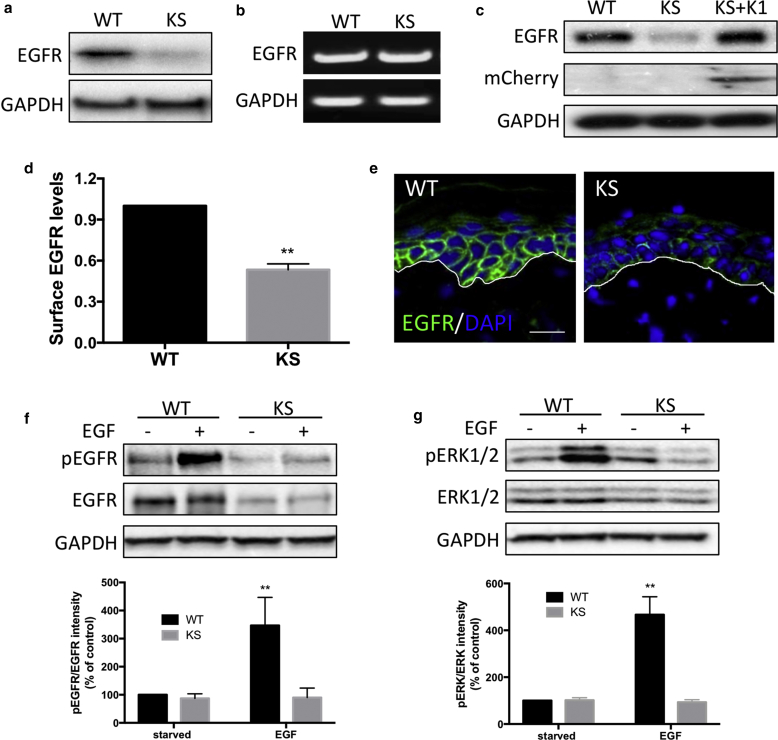
**EGFR levels are reduced in keratinocytes lacking kindlin-1.** (**a, b**) Levels of (**a**) EGFR protein and (**b**) mRNA in WT and KS keratinocytes. (**c**) Western blot of EGFR levels in WT, KS, KS re-expressing mCherry–kindlin-1 cells. (**d**) Quantification of EGFR surface levels in WT and KS cells by FACS. (**e**) Immunostaining of WT and KS skin for EGFR (green) and DAPI (blue). White line indicates dermal-epidermal junction. (**f, g**) Analysis of (**f**) EGFR and (**g**) ERK1/2 phosphorylation in WT and KS cells after 10 minutes of EGF stimulation. GAPDH was used as a loading control for Western blots. Scale bar = 20 μm. Data are means ± standard error of the mean. ^∗∗^*P* < 0.01 by *t* test. EGFR, epidermal growth factor receptor; KS, Kindler syndrome; WT, wild type.

To identify new pathways downstream of kindlin-1, we profiled lysates of keratinocytes from healthy donors (wild type [WT]) and two different KS patients using mass spectrometry. This analysis showed a reduction in protein levels of EGFR in KS keratinocytes, which was verified using Western blotting (Figure 1a). However, no change in mRNA levels of EGFR was detected in KS cells by semiquantitative reverse transcriptase–PCR (Figure 1b). Analysis of normal human lung (16HBE) and breast (MCF10A) epithelial cell lines also showed a reduction of EGFR levels upon small interfering RNA depletion of kindlin-1 (see Supplementary Figure S1a and b online), suggesting a common role for kindlin-1 in regulating EGFR levels in human epithelial cells. Exogenous expression of kindlin-1 in keratinocytes restored EGFR levels (Figure 1c), thereby specifically attributing this phenotype to kindlin-1 expression. Taken together, these findings show a global reduction in EGFR levels when kindlin-1 is absent or depleted. Further analysis by FACS analysis confirmed a reduction in EGFR surface levels in KS keratinocytes (Figure 1d). Moreover, immunostaining of healthy donor and KS patient skin sections showed a striking reduction of EGFR in the basal keratinocytes in KS skin compared with WT skin (Figure 1e).

EGFR regulates a number of signaling pathways, which act to regulate keratinocyte survival, growth, adhesion, and migration (Bakker et al., 2017). To examine the effect of loss of kindlin-1 on EGFR signaling, cells were starved overnight and stimulated with EGF for 10 minutes, and the phosphorylations of EGFR (Figure 1f) and its downstream effector ERK1/2 (Figure 1g) were assessed. As expected, EGFR phosphorylation in response to EGF was significantly reduced in KS keratinocytes, in line with the constitutively lower levels of EGFR in these cells (Figure 1f), with a resulting loss of EGF-dependent ERK1/2 phosphorylation (Figure 1g). To determine whether this loss of EGF responsiveness had an impact on functional cell behavior, we assessed migratory responses to EGF by time lapse microscopy. Data showed that KS cells exhibited higher migration speeds compared with WT cells under starved conditions, as we have shown previously (see Supplementary Figure 1c and d) (Maiuri et al., 2012). Addition of EGF led to increased WT keratinocyte migration rates but had no effect on KS cell speed, confirming a failure to respond to EGF in the absence of kindlin-1. Migration speeds were rescued in KS cells re-expressing mCherry-kindlin-1, confirming that the observed phenotypes were due to loss of kindlin-1 expression (see Supplementary Figure 1d and e). Together, these findings show that kindlin-1–deficient human keratinocytes have reduced EGFR levels, resulting in impaired responses to EGF.

### Kindlin-1 regulates subcellular distribution and dynamics of EGFR

**Figure 2:**
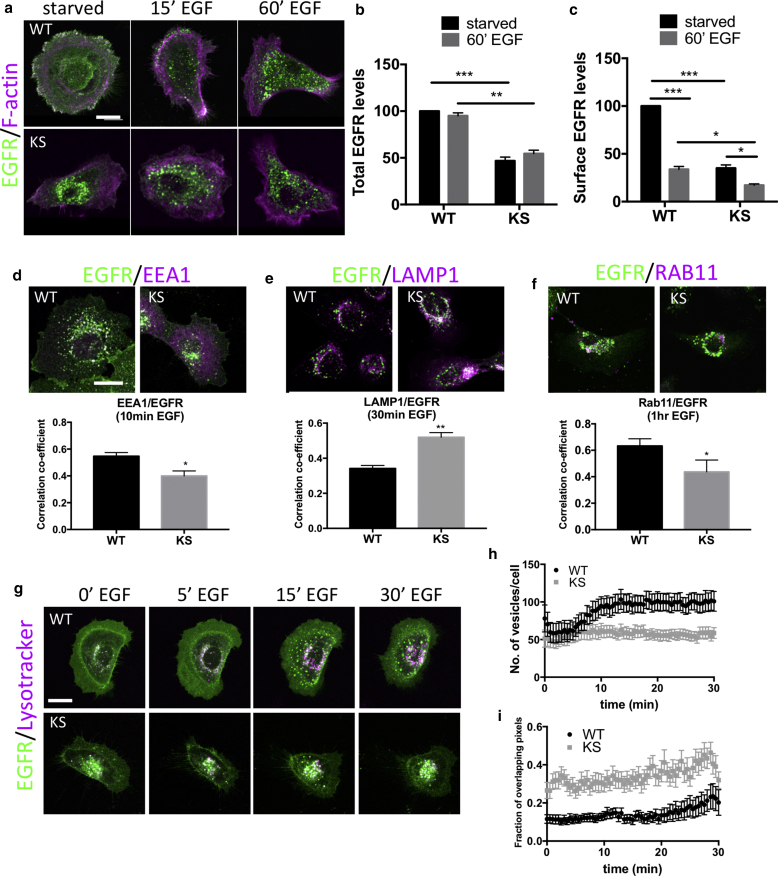
**EGFR localizes to lysosomal compartments in KS cells.** (**a**) Immunostaining of EGFR (green) and F-actin (magenta) and (**b**) quantification of EGFR surface and (**c**) total levels from images in WT and KS cells after EGF stimulation. (**d–f**) Immunostaining and quantification of EGFR (green) localization with (**d**) EEA1, (**e**) LAMP1, or (**f**) Rab11a vesicles (all shown in magenta) after EGF stimulation (10 ng/ml). Graphs beneath images show Pearson correlation coefficient analysis of EGFR and specified compartments. N = 30 cells for each. (**g**) Still images from movies of WT and KS cells expressing EGFR-GFP labeled with LysoTracker Deep Red (Molecular Probes, Eugene, OR) (magenta) after EGF stimulation. (**h**) Quantification of the number of EGFR-positive vesicles and (**i**) EGFR/LysoTracker co-localization from WT and KS movies. N = 25 cells over three independent experiments. Data are all means ± standard error of the mean. ^∗∗∗^*P* < 0.001, ^∗∗^*P* < 0.01, ^∗∗∗^*P* < 0.001 using two-way analysis of variance (**b** and **c**) and *t* test (**d**–**f**). Scale bars = 10 μm throughout. EGFR, epidermal growth factor receptor; hr, hour; KS, Kindler syndrome; min, minute; WT, wild type.

To determine whether the reduced levels of EGFR in KS cells coincided with altered subcellular distribution, we analyzed the localization of EGFR in sparsely plated WT and KS keratinocytes after EGF stimulation. Total and surface levels of EGFR were quantified by measuring the mean fluorescence intensities of either the whole cell area or plasma membrane. Consistent with the Western blot analyses (Figure 1a and f), EGF stimulation did not alter the relative intensity of EGFR in either cell type, but there was a marked reduction in total EGFR levels in KS cells (Figure 2a–c). In starved WT cells, EGFR was localized at the plasma membrane and cytoplasmic compartments, whereas KS cells showed very weak EGFR staining at the plasma membrane with increased accumulation in perinuclear compartments (Figure 2a–c). After EGF treatment, EGFR redistributed from the plasma membrane into perinuclear compartments in WT cells, coincident with reduced EGFR at the plasma membrane (Figure 2c). In contrast, EGFR remained in the perinuclear compartments of KS cells after EGF stimulation (Figure 2c). Kindlin-2 has been shown previously to be expressed at normal levels in KS patients (Lai-Cheong et al., 2008), suggesting that it is not disrupted upon loss of kindlin-1 but also cannot functionally replace kindlin-1 in these cells. However, to determine whether loss of kindlin-1 and resulting EGFR mislocalization could be compensated for overexpression of kindlin-2, WT and KS cells were transfected with GFP–kindlin-2, and total and surface EGFR levels were analyzed by confocal microscopy. Data showed that kindlin-2 overexpression had no effect on EGFR levels or localization in either WT or KS keratinocytes (see Supplementary Figure 1e and f), suggesting that kindlin-2 cannot compensate for loss of kindlin-1 in these cells. Indeed, functional, nonredundant roles for kindlin-1 and -2 have also been suggested in the context of integrin binding in keratinocytes (Bandyopadhyay et al., 2012), further supporting the notion that these proteins have different roles in epithelial cell function.

EGFR is known to undergo endocytosis and, depending on the cell type and EGF concentration, can be recycled back to the plasma membrane or routed for degradation (Bakker et al., 2017). To determine whether kindlin-1 may play a role in regulating EGFR dynamics at the plasma membrane, we analyzed WT and KS cells stably expressing EGFR-GFP after fluorescence recovery after photobleaching at the plasma membrane under growth conditions. Despite expressing lower levels of EGFR, KS cells showed enhanced early recovery profiles compared with WT and reduced T1/2 speed without changing the mobile fraction (see Supplementary Figure S2a online). We confirmed that this effect of kindlin-1 was not due to global changes in clathrin-mediated endocytosis, because transferrin-Texas Red uptake assays showed no differences between WT and KS cells (see Supplementary Figure S2b), indicating that global receptor internalization was unperturbed by the loss of kindlin-1. Inhibition of dynamin activity, but not recycling (through dynasore and primaquine treatment, respectively), resulted in a slower fluorescence recovery T1/2 and reduced EGFR mobile fraction (see Supplementary Figure 2c and d). These data show that loss of kindlin-1 destabilizes EGFR under steady state conditions and that inhibition of EGFR internalization, but not receptor recycling, reduces EGFR dynamics at the plasma membrane.

To determine potential kindlin-1–dependent changes in EGFR subcellular compartmentalization, we used colocalization analysis to study EGFR localization with key endocytic markers at time points after EGFR stimulation: early endosomes (EEA1, 10 minutes), lysosomes (LAMP1, 30 minutes), and recycling endosomes (Rab11a, 1 hour). Pearson correlation analysis showed significantly reduced co-localization between EGFR/EEA1 and EGFR/Rab11 in KS compared with WT cells (Figure 2d and f). In contrast, a significant increase in colocalization between EGFR and LAMP1 was observed in KS cells compared with WT (Figure 2e). To further explore the real-time dynamics of the EGFR-positive compartments after EGF stimulation, we performed live cell imaging on WT and KS cells expressing EGFR-GFP and cherry-Rab11a and labeled with LysoTracker Far Red (Molecular Probes, Eugene, OR), for 30 minutes after EGF stimulation. Upon addition of EGF, EGFR-positive vesicles moved in a retrograde fashion from the plasma membrane into the cell interior, increasing in number and size over time (Figure 2g and h and see Supplementary Movie S1 online). In contrast, EGFR-labeled vesicles in KS cells displayed random movement in the perinuclear region throughout the 30 minutes of observance, with the size and vesicle number remaining largely unaltered (Figure 2g and h, and see Supplementary Movie S2 online). Analysis of overlapping pixels in the EGFR-GFP– and lysotracker-labeled vesicles confirmed the LAMP1 data in fixed cells (Figure 2e), showing a constitutively higher co-localization between EGFR-positive vesicles and lysosomal compartments in KS cells compared with WT cells throughout the period of EGF stimulation (Figure 2i).

### Kindlin-1 regulates cellular EGFR levels by restricting lysosomal degradation of EGFR

**Figure 3:**
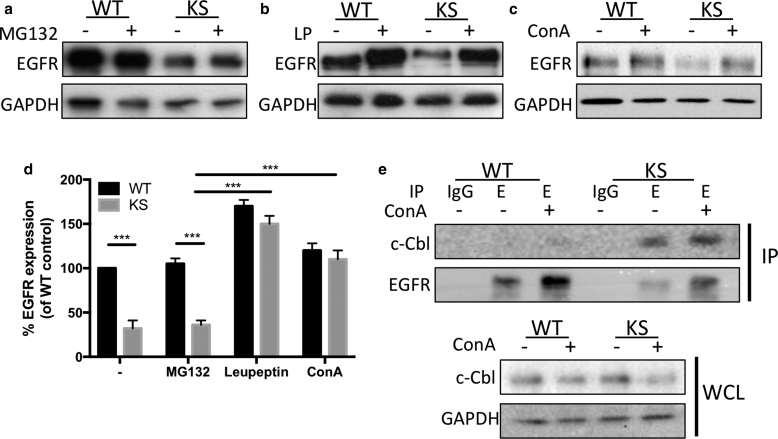
**EGFR is degraded in the lysosome in KS cells through increased Cbl interactions.** (**a–c**) Treatment of WT and KS cells with (**a**) proteasome inhibitor (MG132; 10 μmol/L, 24 hours) or lysosome inhibitors (**b**) leupeptin (100 nmol/L, 24 hours) (**c**) or concanamycin A (100 nmol/L, 24 hours) and analysis of EGFR levels by Western blotting. (**d**) Quantification of Western blots in **a**–**c** from four independent experiments. (**e**) Immunoprecipitation of EGFR from WT and KS cells after DMSO (–) or ConA treatment (100 nmol/L, 24 hours; +) and immunoblotting for c-Cbl. Blots beneath show c-Cbl levels in whole cell lysates. ConA, concanamycin A; EGFR, epidermal growth factor receptor; IP, immunoprecipitation; KS, Kindler syndrome; LP, leupeptin; WT, wild type.

EGFR is subject to ligand-induced degradation via the lysosomal or proteasomal pathways (Singh and Coffey, 2014). Given the increased EGFR within lysosomal compartments in KS cells, we next analyzed whether EGFR was reduced in KS cells because of enhanced protein degradation. Treatment of WT and KS cells with the proteasome inhibitor MG132 did not change EGFR levels in KS cells (Figure 3a and d). However, treatment with lysosomal inhibitors leupeptin or concanamycin A restored EGFR expression in KS cells up to WT levels (Figure 3b–d), suggesting that loss of kindlin-1 leads to increased lysosomal-dependent EGFR degradation. EGFR binds to the E3 ubiquitin ligase c-Cbl in response to EGF, either at the plasma membrane or on early endosomes, which in turn promotes polyubiquitination of EGFR, resulting in degradation (Duan et al., 2003). To determine whether kindlin-1–dependent changes to EGFR altered c-Cbl association with the receptor, we analyzed c-Cbl-EGFR binding by co-immunoprecipitation (IP) in WT and KS cells treated with DMSO or concanamycin A under growth conditions. A dramatic increase in c-Cbl binding to EGFR in KS cell lysates was observed, with or without treatment with concanamycin A (Figure 3e), suggesting that increased constitutive c-Cbl binding in the absence of kindlin-1 may result in increased targeting of EGFR for lysosomal degradation.

### Kindlin-1 directly interacts with EGFR

**Figure 4:**
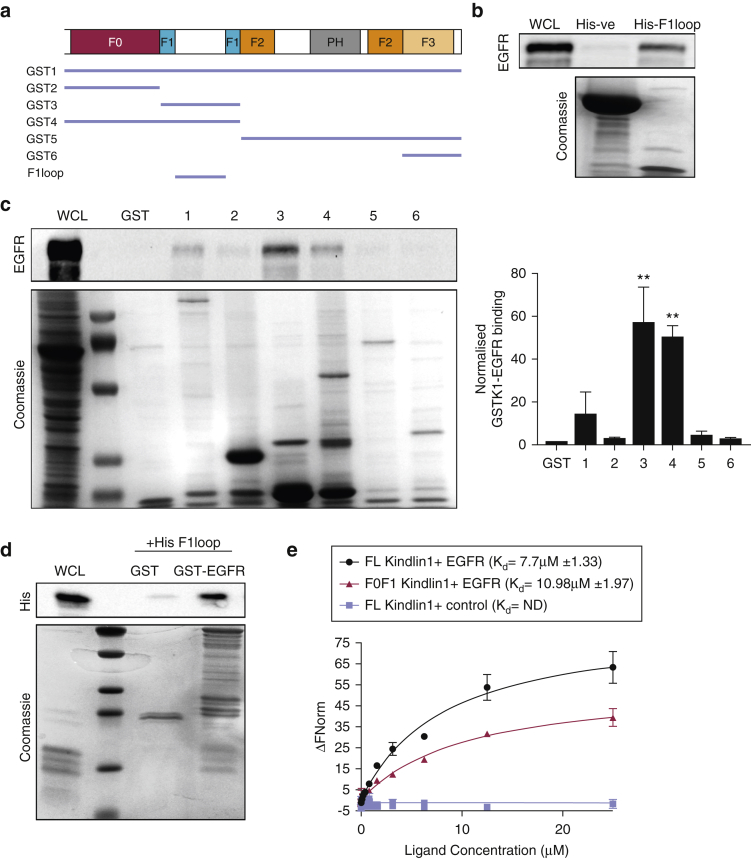
**EGFR directly interacts with kindlin-1 via the F1 loop region.** (**a**) Diagram of GST- and His-tagged kindlin protein domains were used. (**b**) GST pulldown of kindlin-1 domains and immunoblotting for EGFR in WT keratinocytes. Quantification of GST-K1 domains and EGFR interaction are shown in the graph. (**c**) His kinesin light chain (-ve control) or kindlin-1 F1 loop incubated with purified GST-EGFR cytoplasmic domain. (**d**) In vitro binding of GST-EGFR cytoplasmic tail and His-kindlin F1 loop (His1) using GST pulldown. (**e**) MST analysis of full-length (black) or F0F1 (red) kindlin-1 and EGFR cytoplasmic tail interaction. R7–R9 of Talin (blue) was used as a control. All data are means ± standard error of the mean from three independent experiments. ^∗∗^*P* < 0.01 using one-way analysis of variance. EGFR, epidermal growth factor receptor; M, mol/L; MST, microscale thermophoresis; ND, not done; WCL, whole cell lysate.

Kindlin-2 has previously been suggested to directly interact with EGFR through an association with the EGFR kinase domain (Guo et al., 2015). To determine whether kindlin-1 could interact with EGFR, individual domains of kindlin-1 were generated as GST fusion proteins and used to pull out endogenous EGFR from cell lysates (Figure 4a). Full-length GST–kindlin-1 (GST1) bound to EGFR and a consistently strong binding with the F1 domain of kindlin-1 was also observed (GST3) (Figure 4b). The F1 domain contains an unstructured loop that we postulated could be a potential binding region for EGFR (Bouaouina et al., 2012). We tested this hypothesis by expressing a His-tagged F1 loop to capture EGFR from cell lysates. As predicted, the F1 loop bound strongly to EGFR in cell lysates in contrast to the His-kinesin light chain domain that served as a negative control (Figure 4c). To test whether association between kindlin-1 and EGFR was direct, a GST fusion of the EGFR cytoplasmic domain was incubated with His-F1 loop of kindlin-1 in solution. Pulldown of the GST-EGFR cytoplasmic tail showed a strong interaction with the His–kindlin-1 F1 loop (Figure 4d), indicating a direct interaction between the two proteins. Moreover, assessment of binding kinetics between these proteins by microscale thermophoresis (MST) showed a robust interaction between the EGFR cytoplasmic tail membrane proximal region and both full-length and F0F1 domains of kindlin-1 (Figure 4e). Taken together, these data show that kindlin-1 binds directly to the EGFR cytoplasmic domain via the kindlin-1 F1 loop. Moreover, the fact that c-Cbl binding is significantly and constitutively enhanced in cells lacking kindlin-1 (Figure 3e) suggests that binding of kindlin-1 to the EGFR cytoplasmic tail restricts binding of c-Cbl, leading to retention of EGFR at the plasma membrane, enhanced signaling, and reduced degradation.

**Figure 5:**
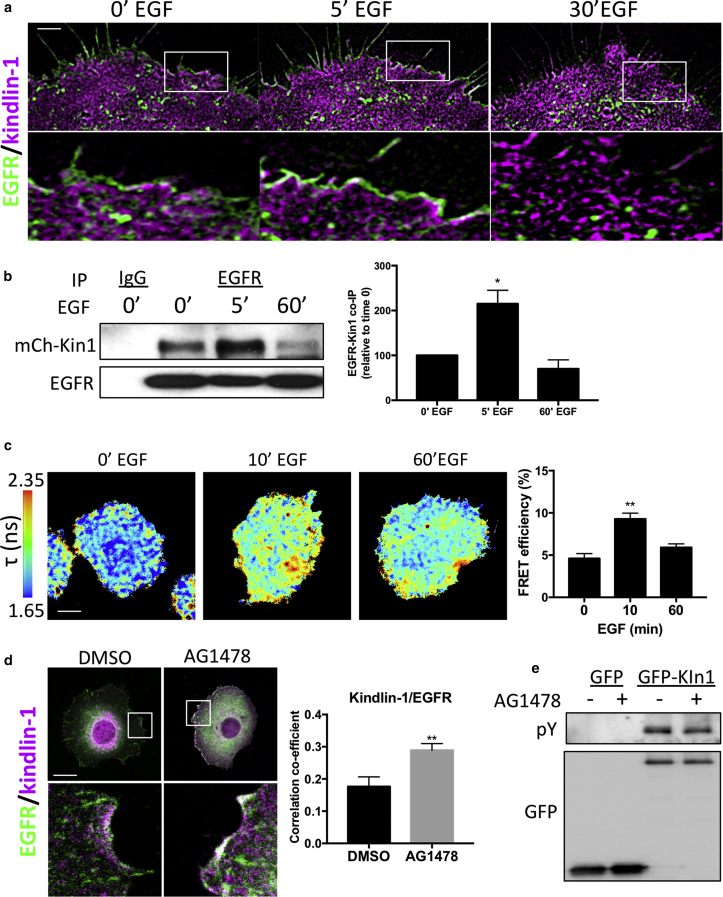
**EGFR–kindlin-1 binding in human keratinocytes is EGF regulated.** (**a**) SIM imaging of EGFR-GFP (green) and mCherry–kindlin-1 (magenta) after EGF stimulation. Inset boxes shown below each time point. (**b**) Immunoprecipitation of EGFR from KS cells re-expressing mCherry–kindlin-1 under starved conditions or after EGF stimulation (10 ng/ml). Graph on right shows quantification of five independent experiments. (**c**) Example lifetime images of KS cells expressing kindlin-GFP and mCherry–kindlin-1 after EGF stimulation. Graph on right shows quantification of 25 cells per condition over three experiments. (**d**) Example images of KS cells re-expressing mCherry–kindlin-1 (magenta) under growth conditions with DMSO or AG1478 treatment, fixed and stained for EGFR (green). Inset boxes shown below. Graph on right shows quantification of co-localization from 30 cells. (**e**) Immunoprecipitation of GFP or GFP–kindlin-1 under same conditions as in **d**, probed for phosphotyrosine (pY) and GFP. ^∗^*P* < 0.05, ^∗∗^*P* < 0.01 using two-way analysis of variance. Scale bars = 1 μm in **a** and 10 μm in **c** and **d**. EGFR, epidermal growth factor receptor; min, minute; SIM, structure illumination microscopy.

To further define when and where kindlin-1 may associate with EGFR in cells, we analyzed their relative subcellular distributions using live-cell structure illumination microscopy superresolution imaging of KS cells expressing mCherry–kindlin-1 and EGFR-GFP. Images and subsequent analysis showed that co-localization between the two proteins occurred within the first 15 minutes of EGF stimulation at the plasma membrane (Figure 5a, and see Supplementary Figure S3a online). We were also unable to detect any kindlin-1 co-localizing with EGFR within endosomes. IP of endogenous EGFR from KS cells re-expressing mCherry–kindlin-1 also showed that kindlin-1 forms a complex with EGFR in a time-dependent manner, with strongest interactions occurring 5 minutes after EGF stimulation and resuming to basal levels by 60 minutes (Figure 5b). However, we were unable to detect kindlin-2 in these immunoprecipitated complexes (see Supplementary Figure S3b), suggesting that the binding of kindlin-1 may be specific in keratinocytes.

Analysis of direct binding between the two proteins using fluorescence lifetime imaging microscopy to analyze fluorescence resonance energy transfer (FRET) also showed a direct interaction between EGFR-GFP and mCherry–kindlin-1 in cells that was increased after 10 minutes of EGF stimulation (Figure 5c). Moreover, kindlin-1–to–EGFR binding was independent of kindlin-1–to–integrin binding, because FRET-fluorescence lifetime imaging microscopy analysis showed strong, constitutive interaction between EGFR-GFP and mCherry–W612Akindlin-1, which is defective in integrin binding (see Supplementary Figure S3c) (Bouaouina et al., 2012, Huet-Calderwood et al., 2014). Further analysis of these cells showed that expression of mCherry–W612Akindlin-1 in KS cells was also able to partially restore the migration response to EGF (see Supplementary Figure S3d), further indicating that kindlin-1–to–EGFR binding plays an important role in control of EGF responses and that this can act at least in part independently of kindlin-1–to–integrin complex formation.

To explore whether EGFR kinase activity regulates kindlin-1–EGFR binding, we assessed the co-localization between endogenous EGFR and GFP–kindlin-1 expressed in KS cells in the presence of either DMSO or AG1478, an EGFR-specific tyrosine kinase inhibitor. Inhibition of EGFR activity resulted in an increase in co-localization between EGFR and GFP–kindlin-1 (Figure 5d), potentially through enrichment of EGFR at the plasma membrane. Finally, because kindlin-2 has previously been suggested to be tyrosine phosphorylated (Liu et al., 2015, Qu et al., 2014), we sought to determine whether the same modification on kindlin-1 could occur through EGFR-mediated signaling. IP analysis showed that GFP–kindlin-1 was tyrosine phosphorylated under basal conditions (Figure 5e). However, treatment with AG1478 had no effect on kindlin-1 tyrosine phosphorylation levels, suggesting that kindlin-1 is constitutively tyrosine phosphorylated in growth conditions and that this does not depend on signals downstream of active EGFR.

In summary, our data show a direct interaction between kindlin-1 and EGFR that acts to restrict c-Cbl–EGFR association and thus protect EGFR from lysosomal degradation. Although our data do not allow us to conclusively state that EGFR-Cbl binding in KS cells is constitutive, our data support the notion that the presence of kindlin-1 is required to ensure correct regulation of the EGFR-Cbl complex. Our proposed model is that binding of kindlin-1 to the EGFR cytoplasmic tail can displace Cbl binding and potentially act to stabilize EGFR at the membrane and subsequently control modulation of EGFR routing to the endo-lysosomal system. Loss of kindlin-1 expression in patients with KS results in lower EGFR levels in the skin and isolated keratinocytes, resulting in loss of EGF-induced signaling and migratory behavior. This newly described function for kindlin-1 is very likely to contribute to the clinical features observed in KS patients in agreement with our recent discovery of an EGFR loss-of-function mutation in patients with skin fragility (Campbell et al., 2014). Based on our data, investigations of strategies to modulate EGFR stability may represent a valid therapeutic avenue for treating skin fragility patients in future.

## Materials and Methods

### Plasmids and small interfering RNAs

GFP–kindlin-1, GFP–kindlin-1W612A, and GFP–kindlin-2 constructs were generously provided by David Calderwood (Yale University, New Haven, CT [Bouaouina et al., 2012, Huet-Calderwood et al., 2014]). EGFR-GFP was provided by Andy Reynolds (AstraZeneca, Cambridge, UK [Reynolds et al., 2003]). EGFR cytoplasmic domain GST fusion constructs were generously provided by Bob Adelstein (National Institutes of Health, Bethesda, MD [Kim et al., 2012]). Murine full-length kindlin-1 and kindlin-1 F0F1 (1-275) were cloned into a pET151 vector (Invitrogen, Waltham, MA). Cherry-kindlin1 and cherry-kindlin1W612A lentiviral constructs have been previously described (Zhang et al., 2016). mCherry-Rab11a was a gift from Patrick Caswell (University of Manchester, Manchester, UK [Caswell et al., 2007]).

### Reverse Transcriptase PCR

RNA extraction from cells was performed using RNAeasy Mini Kit (Qiagen, Hilden, Germany) and reverse transcription of RNA was carried out using RevertAid Reverse Transcriptase (Thermo Fisher Scientific, Waltham, MA), according to the manufacturer’s instructions. Reverse transcriptase–PCR primer sequences are as follows: GAPDH (forward 5′-AGAAGGCTGGGGCTCATTTG-3′, reverse 5′-AGGGGCCATCCACAGTCTTC-3′); kindlin-1 (forward 5′-TCAAACAGTGGAATGTAAACTGG-3′, reverse 5′-TACATGCTGGGCACGTTAGG-3′).

### Cell culture and transfections

Immortalized WT keratinocytes and those from a KS patient (harboring the mutation c.676insC/c.676insC) have both been previously described (Lai-Cheong et al., 2007, Zhang et al., 2016). The original study in which the cells were isolated was conducted according to the principles of the Declaration of Helsinki. All cells were obtained under the St. Thomas Hospital Ethics Committee-approved project “Molecular Basis of Inherited Skin Disease” (07/H0802/104) after participating individuals gave written, informed consent. Both cell lines were grown in serum-free keratinocyte growth medium containing EGF and bovine pituitary extract (Gibco, Waltham, MA) and supplemented with penicillin and streptomycin. HEK293T cells were cultured in DMEM supplemented with penicillin and streptomycin, l-glutamine, and 10% fetal bovine serum. 16HBE cells were grown in minimum essential media supplemented with penicillin and streptomycin, l-glutamine, and 10% FBS. MCF10A cells were cultured in DMEM supplemented with penicillin and streptomycin, l-glutamine, 5% horse serum, EGF (20 ng/ml), hydrocortisone (0.5 μg/ml), cholera toxin (100 ng/ml) and insulin (10 μg/ml). HEK 293T transfections were performed using polyethylenimine transfection reagent at a 1:7 ratio of DNA to polyethylenimine reagent. Keratinocyte transfection of plasmids was carried out using Attractene transfection reagent (Qiagen), and all small interfering RNA transfections were performed using Dharmofect transfection reagent (Dharmacon, Lafayette, CO), in accordance with the manufacturer’s instructions. Inhibitors were all purchased from Sigma and used at the following concentrations: leupeptin (100 nmol/L, 4 hours), MG132 (20 μmol/L, 4 hours), concanamycin A (100 nmol/L, 16 hours), dynasore (80 μmol/L, 1 hour), primaquine (100 μmol/L, 1 hour), and AG1478 (5 nmol/L, 1 hour).

### Antibodies

Primary antibodies used were EGFR (for Western blot: Cell Signaling Technologies, Danvers, MA; for IP: Cell Signaling Technology; and for immunofluorescence: Santa Cruz Biotechnology, Dallas, TX), kindlin-2 (Abcam, Cambridge, UK), phospho-EGFR Y1173 (Cell Signaling Technology), phospho-tyrosine (4G10; Millipore, Billerica, MA), GFP (Roche, Basel, Switzerland), HA (Cell Signaling Technology), c-Cbl (Cell Signaling Technology), GAPDH (Genetex, Taiwan, China), HSC70 (Santa Cruz Biotechnology), EEA1 (Cell Signaling Technology), LAMP1 (Cell Signaling Technology), phospho-ERK1/2 (Cell Signaling Technology), ERK1/2 (Cell Signaling Technology), GST (Sigma-Aldrich, St. Louis MO), His (horseradish peroxidase conjugate; Millipore). All anti-species horseradish peroxidase-conjugated secondary antibodies were from Dako (Carpinteria, CA), and all AlexaFluor conjugated antibodies were from Molecular Probes. Other reagents and suppliers were Phalloidin AlexaFluor (Molecular Probes), Hoechst (Sigma-Aldrich), lysotracker deep red (Molecular Probes), transferrin Texas Red (Thermo Fisher Scientific).

### GST- and His-tagged protein purification

Protein production was induced in *Escherichia coli* BL21 bacterial strains with IPTG (100 μmol/L) overnight at 18°C. For GST-tagged proteins, bacterial pellets were resuspended in 50 mmol/L Tris, 300 mmol/L NaCl, pH 8.0 in the presence of protease inhibitors; sonicated; and cleared by centrifugation. The protein solution was then incubated with glutathione Sepharose beads (GE Healthcare, Little Chalfont, UK) for 2 hours at 40°C followed by three washes in 50 mmol/L Tris, 300 mmol/L NaCl, pH 8.0 with 2 mmol/L β-mercaptoethanol. The GST-tagged proteins were either left bound to the beads (for GST pulldown experiments) or eluted with glutathione solution (50 mmol/L Tris, 300 mmol/L NaCl, 40 mmol/L glutathione, pH 8.0) and dialyzed overnight. For His-tagged proteins, bacterial pellets were resuspended in His lysis buffer (25 mmol/L HEPES pH 8.0, 500 mmol/L NaCl, 10 mmol/L imidazole) containing protease inhibitors, sonicated, and cleared by centrifugation. Nickel NTA beads (Qiagen) were incubated with the protein solution for 2 hours at 4°C, followed by three washes in lysis buffer containing 50 mmol/L imidazole. The His-tagged proteins were eluted from the beads with lysis buffer containing 250 mmol/L imidazole, followed by overnight dialysis. For MST analysis, standard nickel-affinity chromatography was used to purify the His-tagged recombinant proteins, as described previously (Banno et al., 2012). Purified samples were analyzed by SDS-PAGE on a 10% gel and stained with Coomassie blue. Protein concentrations were determined using the respective extinction coefficients at 280 nm calculated using ProtParam (https://web.expasy.org/protparam/).

### GFP traps, IP, and Western blotting

Cells were lysed in cold lysis buffer (50 mmol/L Tris-HCL, pH 7.4; 200 mmol/L NaCl, 1% NP-40, 2 mmol/L MgCl_2_, 10% glycerol) containing protease inhibitors and phosphatase inhibitors, and lysates were cleared by centrifugation. For GFP traps, the cleared lysates were incubated with GFP trap beads for 2 hours at 4°C, followed by three washes in lysis buffer. For other IPs, cleared lysates were incubated with either antibody- or species-matched IgG overnight and then incubated for 2 hours with protein A/G beads (preblocked with 0.2% bovine serum albumin). Beads were washed three times in lysis buffer and resuspended in sample buffer, boiled for 10 minutes, and resolved on a 12% SDS-PAGE gel. For mass spectrometry analysis, WT and KS lysates were resolved on 10% SDS-PAGE gels and silver stained, and identified bands were excised and sent for processing and analysis to Aberdeen Proteomics (University of Aberdeen, School Medical Sciences, Aberdeen, MD).

### Flow cytometry

FACS analysis was performed as previously described (Worth et al., 2010). Briefly, cells were scraped with phosphate buffered saline (PBS) and fixed with 4% paraformaldehyde for 20 minutes. Cells were then blocked in PBS containing 2% bovine serum albumin, incubated with primary antibody for 90 minutes, washed three times, and then incubated with secondary antibody for 45 minutes, followed by three washes and final resuspension in PBS. As a negative control, a secondary antibody-only sample was used, and fluorescence reading from this was used to indicate background fluorescence values. Data were analyzed using FlowJo software (FlowJo, Ashland, OR).

### Immunofluorescence and microscopy

Cells were plated onto coverslips or optical plastic-bottom dishes coated with human fibronectin (10 ng/ml, Millipore). After respective treatments, cells were either used for live cell imaging or fixed using 4% paraformaldehyde (paraformaldehyde/PBS) for 10 minutes, washed with PBS, and then permeabilized with either methanol at –20°C (for endocytic markers) or 0.2% Triton-X/PBS for 5 minutes. Coverslips were then washed with PBS and blocked with 5% bovine serum albumin/PBS for 30 minutes. The primary and secondary antibodies were diluted in 5% bovine serum albumin/PBS and incubated for 1 hour each at room temperature, with PBS washes between the antibody incubations. Coverslips were mounted onto slides using FluorSave reagent (Calbiochem, San Diego, CA). Cell images, fixed and live, were acquired on the Nikon A1R confocal microscope (Nikon Instruments, Kingston, UK) at excitation wavelengths of 405 nm, 488 nm, 543 nm, and 633 nm using a PlanApo VC 60× Oil NA 1.4 objective.

### Random migration assay

Cells were seeded onto 12-well plates, starved overnight in Opti-MEM (Gibco), and then stimulated with EGF (10 ng/ml) before imaging, which was performed on an Olympus (Tokyo, Japan) IX71 microscope using an automated x,y,z scanning stage (Ludl, Hawthorne, NY). Phase contrast images were acquired using a 10× N-Achroplan NA 0.25 objective, and images were taken every 10 minutes for 16 hours using a Sensicam (PCO Cook, Kelheim, Germany) charge-coupled device camera and AQM acquisition software (Andor Bioimaging, Belfast, UK). Single, nondividing cells from the time-lapse movies were then tracked using IQ Tracking Software (Andor Bioimaging). The generated position coordinates for each cell per frame were subject to motion analysis using Wolfram Mathematica 6 notebooks (Wolfram, Champaign, IL) to obtain speed measurements.

### Fluorescence recovery after photobleaching analysis

Fluorescence recovery after photobleaching experiments were performed on cells stably expressing EGFR-GFP. Live cell images were acquired at 5 seconds per frame for three frames, followed by photobleaching of a circular region of interest of 25 pixels in diameter near the cell leading edge using a 488-nm laser at 100% power. Images were acquired for a further 3 minutes at 5 seconds per frame. The rate of fluorescence recovery was calculated by measuring the fluorescence intensity of the region of interest over time. The fluorescence recovery values were corrected for overall fading across the entire image during the imaging period and were represented as a percentage of the pre-bleached values (the average values of the first three frames), which represented the 100% fluorescence signal. Values were fitted to a mono-exponential equation from which the T1/2 and the percentage mobile fraction (plateau) values were determined.

### FRET analysis

FRET efficiency was quantified from KS keratinocytes expressing donor and acceptor fluorophores by measuring time domain fluorescence lifetime with a multiphoton microscope system (TE2000, Nikon). Briefly, cells were fixed in 3.6% formaldehyde for 15 minutes, permeabilized with 0.1% Triton, and quenched with 1 mg/ml sodium borohydride for 10 minutes at room temperature. Cells were mounted or immunostained for flag detection. Fluorescence lifetime was measured as described previously (Zanet et al., 2012), and histogram data show mean FRET efficiency from denoted numbers of cells per condition in three independent experiments using TRI2 analysis software (Paul Barber, University of Oxford, Oxford, UK).

### Image analysis

All images were analyzed using Fiji (https://imagej.net/Fiji) unless otherwise stated. For quantification of surface levels and total levels of EGFR, images were manually thresholded, and intensity values were calculated per cell area and normalized to the control cells in that sample set. Co-localization analysis was performed on the fixed confocal images using the Coloc2 plugin in Fiji, by either drawing a region of interest around the cells to measure total co-localization within the cell or drawing a 10 pixels wide line along the leading edge to measure co-localization at the leading edge. A Python script was created in-house for the analysis of vesicle size, number, and EGFR-LAMP1 co-localization. Vesicles were identified by wavelet-filtering the images, followed by thresholding and watershed segmentation, using a similar process to that described by Izeddin et al. (2012). After segmentation, vesicle analysis proceeded using a similar methodology as previously published (Rizk et al., 2014).

### Microscale thermophoresis

Recombinantly expressed kindlin-1 constructs were coupled to an equimolar amount of RED-tris-NTA NT-647 dye (NanoTemper Technologies, München, Germany) via its N-terminal 6×His-Tag in a one-step coupling reaction (Bartoschik et al., 2018). Titrations were performed in PBS (137 mmol/L NaCl, 27 mmol/L KCl, 100 mmol/L Na_2_HPO_4_, 18 mmol/L KH_2_PO_4_) using a constant 50-nmol/L concentration of RED-tris-NTA–coupled kindlin, with increasing concentration of synthetic EGFR peptide (residues 668-711: CMRRRHIVRKRTLRRLLQERELVEPLTPSGEAPNQALLRILKETE) and final volume of 20 μl. Prepared samples were filled into Monolith NT.115 Capillaries (NanoTemper). Measurements were recorded on a Monolith NT.115 at 25°C, excited under red light, medium MST power, and 40% excitation power. The data were analyzed using MO Affinity Analysis software (NanoTemper) and fitted using the *K*_d_ fit model.

### Statistical analysis

All statistical tests were performed using either t tests or analysis of variance in GraphPad Prism (GraphPad, La Jolla, CA. All data represent at least three independent experiments. Statistically significant results were taken as *P* < 0.05, and significance values were assigned in specific figures/experiments as shown.

## Conflict of Interest

The authors state no conflict of interest.
