# Supplementary material for: Kindlin-1 Regulates Epidermal Growth Factor Receptor Signaling
Source: J Invest Dermatol. 2019 Feb;139(2):369–79. doi: 10.1016/j.jid.2018.08.020 (PMC6345584; doi:10.1016/j.jid.2018.08.020)
Supplement: Supplementary Figures S1–S3 and Supplementary Movies S1 and S2 [file mmc1.pdf]

Supplementary Figure S1

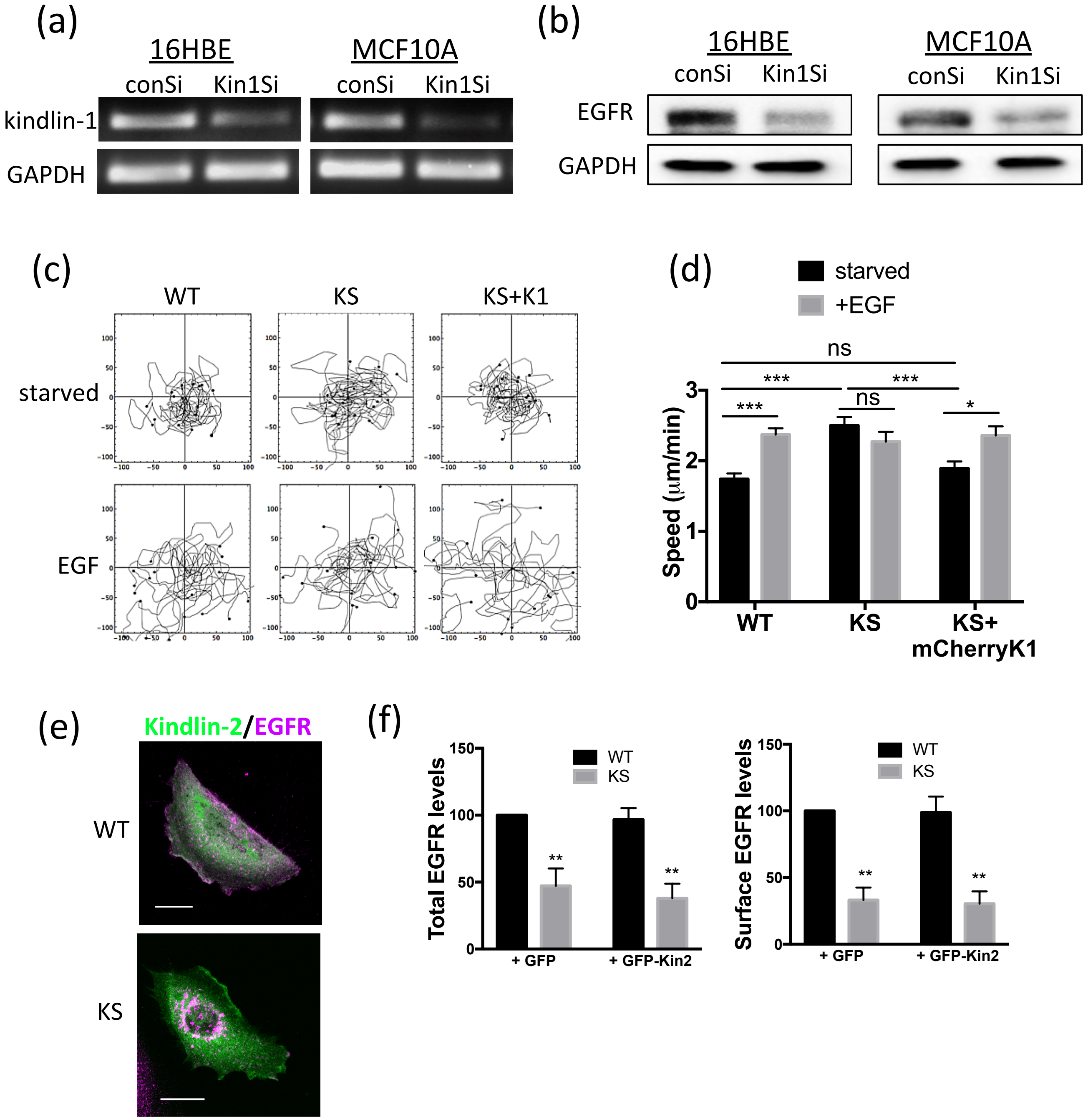

Supplementary Figure 1: Loss of kindlin-1 results in loss of migratory response to EGF
(a) RT-PCR analysis of kindlin-1 in human bronchial epithelial (16HBE) and breast epithelial (MCF10A) cells treated with control of kindlin-1 siRNAs (b) Western blot of cells in (a) for EGFR. GAPDH was used as a loading control. (c) Tracks of WT and KS cells undergoing random migration under starved and EGF stimulated conditions 10ng/ml; 16 hours. (d) Quantification of random migration speeds from cells in (c). Data are means ± SEM; *P<0.05, ***P<0.001, using two-way ANOVA, Sidak's multiple comparison test. (e) Representative images of WT or KS cells expressing Kindlin2-GFP stained for EGFR (magenta). Scale bars, 10 μm. (f) Quantification of total (left graph) and cell surface (right graph) EGFR levels quantified from images of cells expressing GFP or GFP-kindlin-2 either fixed and permeabilised (total) or unpermeabilised (surface) before staining.

Supplementary Figure S2

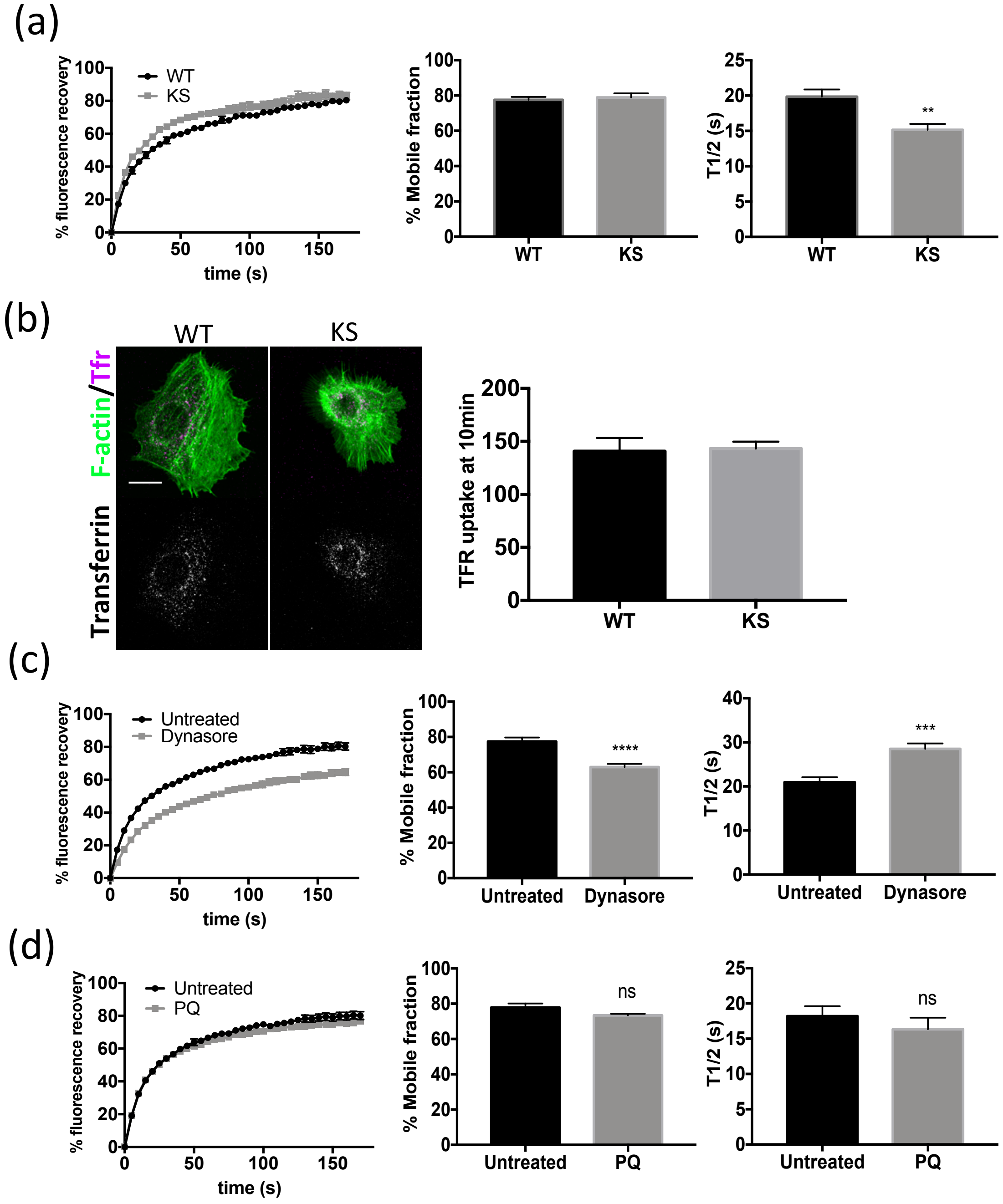

Supplementary Figure 2: EGFR is destabilised at the plasma membrane in KS cells
(a) FRAP analysis of EGFR dynamics in WT and KS cells expressing EGFR-GFP. EGFR-GFP recovery under growth conditions was analysed in WT and KS cells. Quantitation of T1/2 and mobile fractions were performed by analysis of fluorescence recovery curves. (b) Example images of WT and KS cells incubated with 50 µg/ml Tfr-Texas-Red (magenta) for 10min and fixed, stained for F-actin (green). Graph on right shows Tfr uptake quantified by measuring the mean fluorescence intensity of the Texas-Red signal for each cell using the phalloidin-A488 staining to determine the cell area. (c,d) FRAP analysis of EGFR dynamics in WT cells treated for 1hr with (c) 80 nM dynasore or (d) 100 µM Primaquine (PQ). DMSO or water served as controls for the dynasore and PQ treatments, respectively. Quantitation of T1/2 and mobile fractions were performed by analysis of fluorescence recovery curves. All data are means ± SEM from 3 independent experiments; ***<P:0.001, using T-test. Scale bar, 10µm.

Supplementary Figure S3

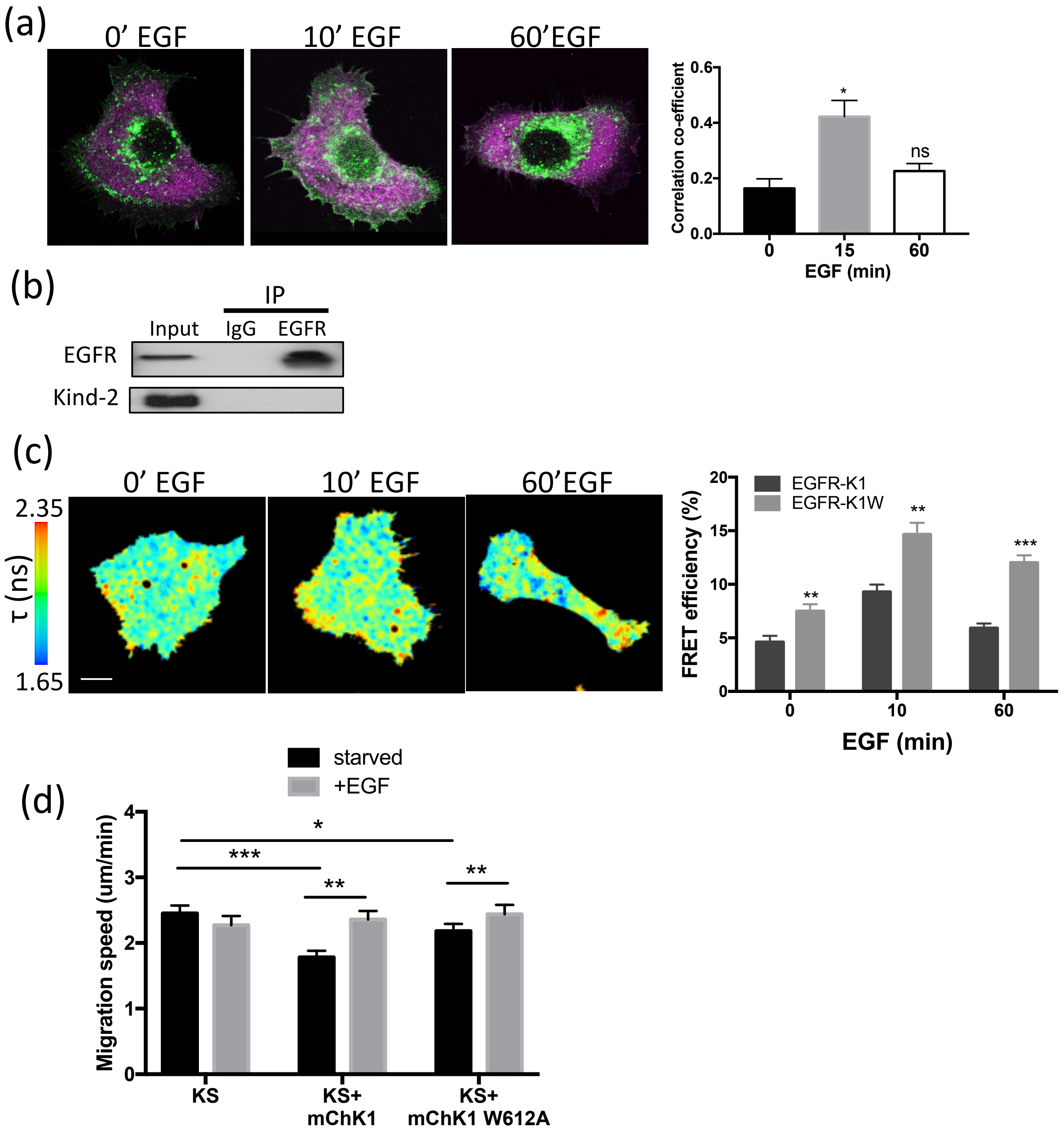

Supplementary Figure 3: Kindlin-1-EGFR binding does not require kindlin-1-integrin binding
(a) Example images of KS cells re-expressing cherry-kindlin-1 (magenta) and EGFR-GFP (green) starved or following EGF stimulation (10ng/ml). Graph on right shows quantification of colocalisation by Pearsons Correlation co-efficient from 30 cells. (b) Representative western blots of WT keratinocyte lysates immunoprecipitated for EGFR (or IgG as a control) and probed for EGFR or kindlin-2. (c) Representative lifetime images of FRET between EGFR-GFP and cherry-kindlin1W612A interaction in KS following EGF stimulation. Graph on right is mean FRET efficiency from 24 cells per condition. All data are means ± SEM from 3 independent experiments; Scale bars, 10µm. (d) Quantification of random migration speed of KS cells versus KS cells expressing WT or W612A cherry-kindlin-1. Cells were either starved, or starved and treated with EGF (10ng/ml). ***<P:0.001, **P<0.01,*P<0.05, using one-way ANOVA, Tukey's multiple comparison test.

Supplementary Movies S1 and S2: Kindlin-1 is required for subcellular distribution of EGFR

Example time lapse confocal microscopy movies of EGFR-GFP (green) and lysotracker-far red (magenta) expressed in normal human keratinocytes (Movie 1) or KS cells (movie 2) following EGF stimulation (10ng/ml) over 30 minutes. Quantification from multiple similar movies is shown in Figures 2 G-I.
